# Label-free quantitative proteomic analysis of *M*. *longissimus dorsi* from cattle during dietary restriction and subsequent compensatory growth

**DOI:** 10.1038/s41598-020-59412-6

**Published:** 2020-02-13

**Authors:** Yvonne Mullins, Kate Keogh, David A. Kenny, Alan Kelly, Padraig O’ Boyle, Sinéad M. Waters

**Affiliations:** 1 Animal and Bioscience Research Department, Animal and Grassland Research and Innovation Centre, Teagasc, Grange, Dunsany, Co., Meath, Ireland; 2 School of Agriculture and Food Science, University College Dublin, Belfield, Dublin, 4 Ireland; 3 Animal and Bioscience Research Department, Animal and Grassland Research and Innovation Centre, Teagasc, Athenry, Co., Galway, Ireland

**Keywords:** Proteomic analysis, Proteomics

## Abstract

Compensatory growth (CG) is a naturally occurring physiological process whereby an animal has the ability to undergo enhanced growth following a period of restricted feeding. This studies objective was to identify key proteins involved in the expression of CG. Forty Holstein Friesian bulls were equally assigned to one of four groups. R1 and R2 groups were subjected to restricted feed allowance for 125 days (Period 1). A1 and A2 animals had *ad libitum* access to feed in Period 1. Following Period 1, all animals from R1 and A1 were slaughtered. Remaining animals (R2 and A2) were slaughtered following *ad libitum* access to feed for successive 55 days (Period 2). *M*. *longissimus dorsi* samples were collected at slaughter from all animals. Proteins were isolated from samples and subjected to label-free mass spectrometry proteomic quantification. Proteins which were differentially abundant during CG (n = 39) were involved in cellular binding processes, oxidative phosphorylation and mitochondrial function. There was also evidence for up regulation of three pathways involved in nucleotide biosynthesis. Genetic variants in or regulating genes pertaining to proteins identified in this study may hold potential for use as DNA based biomarkers for genomic selection of animals with a greater ability to undergo CG.

## Introduction

Compensatory growth (CG) or catch up growth can be defined as a physiological process through which an animal’s growth rate accelerates upon re-alimentation following a period of under nutrition, enabling an animal to reach its genetically pre-determined growth potential^1^. In beef production systems, feed accounts for up to 80% of the total direct costs incurred^2^. Therefore, any method by which these costs can be reduced, without impacting on overall animal performance, would positively influence on farm profitability. The exploitation of the CG phenomenon is a common practice in beef production systems, but particularly in pastoral systems such as in Ireland and has proven useful in reducing over winter animal feed costs^3^. This naturally occurring biological process is also utilised in worldwide beef production systems in ‘stocker’ or ‘backgrounding’ programs^4^. Additionally during CG, animal’s exhibit enhanced feed efficiency, making it a highly desired trait^5,6^. Due to the usefulness of this trait a number of studies have investigated the physiological^5–10^, as well as the molecular control^11–18^ of CG in cattle.

Skeletal muscle has a great economic value accounting for up to 50% of the total body weight of an animal and is in the order of 25% of basal metabolic expenditure^19,20^. This tissue also plays a major role in energy homeostasis. Therefore skeletal muscle (*M*.*longissimus dorsi*) was selected as the target tissue for the current study. Transcriptional profiles have shown that both dietary restriction and subsequent CG are associated with greater expression of genes and pathways involved in metabolism, cellular function, energy production and cellular reorganization^13–15^. However, although transcriptomic data provide useful information on the underlying biology regulating CG, it does not imply that alterations evident at the RNA level are translated into changes at the functional protein level^21^. In almost every organism that has been studied, mRNA transcript levels only partially predict associated protein abundance, usually only explaining 33–66% of the variation in steady-state protein levels^22^. This is due to regulatory processes occurring after the mRNA is formed such as post-transcriptional regulation, translational regulation and protein degradation^21^. Therefore, proteomic studies are an important step towards a more detailed understanding of complex biological systems^23^ and are an indication of differentially expressed genes having a functional role at the phenotype level, which will ultimately contribute to the identification of biomarkers associated with complex traits such as CG^24,25^.

A number of studies have investigated the differences in proteomic profiles in muscle tissue of livestock species^23^ particularly in relation to meat quality characteristics such as muscle differentiation and growth, carcass composition, fat deposition^26–28^, and pre slaughter stress^29^. In addition, a study from our own group by Keady *et al*. (2013) has shown differential muscle proteome expression across bovine breeds varying in genetic merit for carcass weight^24^. However, to the knowledge of the authors, to date, there has been only one such proteomic assessment in relation to CG in cattle^30^. Almeida *et al*.^30^ examined the effects of continuous and discontinuous growth paths on protein abundance in Alentejana bulls using global proteomics. In this study the bulls were allowed to undergo CG for 9 months, until they reached 24 months of age at which point the tissue was sampled. However the peak of CG has been shown to occur up to 2 months into re-alimentation, after-which growth rates start to decline^1,5,7^. Thus, there is no information available in the literature on differential proteomic expression in muscle tissue during the critical CG period following a return to an *ad libitum (ad lib)* diet.

Thus the objective of this study was to conduct a global proteomics approach to quantify variation in protein abundance between cattle undergoing dietary restriction and subsequent CG in comparison to cattle that were continuously fed  *ad lib* or fed a restricted diet, providing an enhanced overview of the biological control of this economically important trait at the peak of its expression.

## Results

### Live weight changes

Details of animal live weights, feed intake and animal performance have been previously published for all animals involved in this study by Keogh *et al*.^5^. At the beginning of Period 1 animals in the R1 group had an average weight of 372 kg (6.84 SEM). The A1 group had an average body weight of 370 kg (6.84 SEM). At the end of differential feeding in Period 1, a 161 kg body-weight difference was evident between the R1 and A1 groups with the R2 group weighing on average 442 kg (6.84 SEM) at the beginning of Period 2 and the average body of the A2 group at 603 kg (6.84 SEM) Following 55 days of *ad lib* feeding for both groups (R2 and A2) in Period 2, the body-weight difference between the groups was reduced to 84 kg. The R2 animals had an average bodyweight of 594 kg (7.95 SEM) and the A2 group having an average bodyweight of 678 kg (7.95 SEM). Therefore in Period 1 bulls in the R1 group had a body-weight gain of 0.6 kg/day and animals in the A1 group has an average daily gain (ADG) of 1.9 kg/day. During Period 2, the previously restricted R2 animals gained 2.5 kg/day compared to the A2 group which gained 1.4 kg/day (P < 0.001). Feed intake was lower in R1 animals compared with A1 animals during Period 1 (P < 0.001). During Period 2, there was no difference observed in feed intake between treatment groups (R2 and A2) (P > 0.05). However when expressed as a proportion of body-weight, feed intake was greater in R2 animals during re-alimentation whilst undergoing CG compared to A2 animals during period 2 (P< 0.001). The bulls in the R2 group showed enhanced growth upon re-alimentation and thus displayed a level of CG as a result. Graphical representation of this data is available in the published work of Keogh *et al*.^5^. The extent of a CG response may be quantified through the ‘compensatory index’. The CG index is calculated as the ratio of the difference between body-weight variation at the end of restricted and CG periods, respectively, relative to the variation at the end of restricted growth alone^1^. The R2 group of animals used in this study displayed a CG index of 48% after only 55 days of re-alimentation.

### Protein identification

Protein identification and quantification was performed using MaxQuant v1.6.2.3. Four experimental comparisons were used: i) R1 (Restricted feeding for 125 days) vs. A1 (*ad lib* feeding for 125 days); ii) R2 (Restricted feeding for 125 days and *ad lib* feeding for 55 days); vs. A2 (*ad lib* feeding for 125 and 55 days); iii) A2 vs. A1; iv) R2 vs. R1.

**Figure 1:**
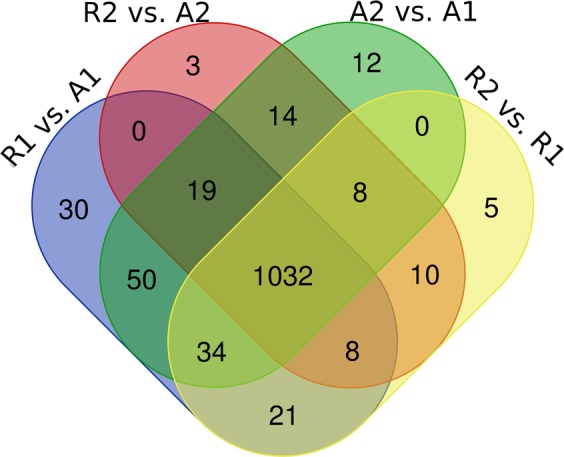
Venn diagram displaying the numbers of proteins identified in *M*.*Longissimus dorsi* of Holstein Friesian bulls in four comparisons: (i) R1 (Restricted feeding for 125 days) vs. A1 (*ad lib* feeding for 125 days); (ii) R2 (Restricted feeding for 125 days and *ad lib* feeding for 55 days); vs. A2 (*ad lib* feeding for 125 and 55 days); (iii) A2 vs. A1; iv) R2 vs. R1.

A total of 1194 (2.9% false positives) proteins were identified in the R1 vs. A1 experiment. Of these 1194 quantified proteins, 4 were identified as differentially abundant proteins (DAPs) (p < 0.05, FC > 1.5). These 4 DAPs are listed in Table 1. Within the group of DAPs, 2 proteins were down-regulated and 2 proteins were up-regulated in the R1 group when compared to the A1 group. The lowest fold change (FC) was observed for Serpin H1 (SERPINH1), a collagen-binding protein (−3.90) and the highest FC of 3.40 was observed for RNA binding motif protein 3 (RBM3). In the R2 vs. A2 analysis, 1094 (3.1% false positives) proteins were quantified. Of these quantified proteins, no proteins were acknowledged as DAPs (p < 0.05, FC > 1.5 or <−1.5) when comparing the two groups. The total number of proteins with at least 2 peptides and a maximum of 5 missing values per protein which could be identified were 1118 in the R2 vs. R1 experiment (3% false positives). Of the 1118 quantified proteins, 39 were identified as DAPs between the two groups (Table 2). Of these DAPs, 23 proteins were down-regulated and 16 were up-regulated in the R2 group compared to the R1 group. The lowest FC (−3.73) among the DAPs was found for ATP synthase subunit d, mitochondrial (ATP5H), and the highest FC (5.42) was for Transketolase (TKT). Lastly in the A2 vs. A1 analysis, 1169 (3% false positives) proteins were identified. Of these 1169 identified proteins, 1 protein was identified as a DAP. The DAP in this comparison was Pyruvate dehydrogenase protein X component (PDHX) with a FC of −2.63 in the A2 group compared to the A1 group with a p-value of 0.003. Figure 1 illustrates the number of proteins identified in each comparison.

**Table 1:** List of differentially regulated proteins identified in Holstein Friesian bulls fed a restricted diet for 125 days (R1) compared to Holstein Friesian bulls fed *Ad libitum* for 125 days (A1).

Accession	Peptide count	Protein name	Gene name	P-value (Bonferonni corected)	Fold change
tr|F1MNT4	24	Laminin subunit beta 1	*LAMB1*	0.004	2.14
tr|F6RBQ9	2	RNA binding motif protein 3	*RBM3*	0.004	3.40
sp|Q2KJH6	14	Serpin H1	*SERPINH1*	0.011	−3.90
tr|G3X6N3	46	Serotransferrin	*TF*	0.020	−2.16

**Table 2:** List of 39 differentially regulated proteins between Holstein Friesian bulls fed a restricted diet for 125 days and subsequently fed *ad libitum* for 55 days (R2) and restricted fed Holstein Friesian bulls for 125 days (R1).

Accession	Peptide count	Protein name	Gene name	P-value (Bonferonni corected)	Fold change
sp|Q6B855	15	Transketolase	*TKT*	0.03	5.42
sp|Q2KJH6	14	Serpin H1	*SERPINH1*	0.01	4.76
tr|F1MNV7	2	N6-adenosine-methyltransferase non-catalytic subunit	*METTL14*	0.04	3.51
tr|F1N650	12	Annexin	*ANXA1*	0.02	3.42
tr|A7Z018	2	RCC1 protein	*RCC1*	0.04	3.37
tr|Q3SZZ9	19	Fibrinogen gamma-B chain	*FGG*	0.01	3.29
tr|E1BP87	170	Myosin heavy chain 4	*MYH4*	0.04	3.20
sp|Q2NKY7	4	Septin-2	*SEPT2*	0.02	3.10
tr|A6QQ11	6	PGM2 protein	*PGM2*	0.00	3.07
sp|Q5E9F5	9	Transgelin-2	*TAGLN2*	0.01	2.86
tr|F1MBI1	4	Proteasome subunit beta type	*PSMB7*	0.04	2.82
tr|G3X6N3	46	Serotransferrin	*TF*	0.00	2.35
sp|Q2KIT0	4	Protein HP-20 homolog	*MGC137014*	0.03	2.24
sp|Q3T0V7	4	Endothelial differentiation-related factor 1	*EDF1*	0.03	2.14
sp|P55859	12	Purine nucleoside phosphorylase	*PNP*	0.01	2.12
sp|Q3SYU2	37	Elongation factor 2	*EEF2*	0.01	1.64
sp|P20004	40	Aconitate hydratase, mitochondrial	*ACO2*	0.05	−1.73
tr|Q3SWX4	9	Glioblastoma amplified sequence	*NIPSNAP2*	0.05	−1.80
sp|P11179	11	Dihydrolipoyllysine-residue succinyltransferase component of 2-oxoglutarate dehydrogenase complex, mitochondrial	*DLST*	0.02	−1.90
tr|G1K1X0	19	Cytochrome b-c1 complex subunit 1, mitochondrial	*UQCRC1*	0.04	−1.93
sp|Q3T0R7	12	3-ketoacyl-CoA thiolase, mitochondrial	*ACAA2*	0.05	−2.01
sp|P11966	12	Pyruvate dehydrogenase E1 component subunit beta	*PDHB*	0.01	−2.02
tr|E1BDK6	39	Laminin subunit beta-2 precursor	*LAMB2*	0.04	−2.09
tr|F1MN74	15	Isocitrate dehydrogenase [NAD] subunit, mitochondrial	*IDH3A*	0.01	−2.14
sp|P00428	8	Cytochrome c oxidase subunit 5B, mitochondrial	*COX5B*	0.01	−2.27
sp|P04394	10	NADH dehydrogenase subunit II	*NDUFV2*	0.04	−2.35
tr|F1MD77	37	Laminin subunit gamma 1	*LAMC1*	0.00	−2.38
sp|Q02380	3	NADH dehydrogenase 1 beta subcomplex subunit 5	*NDUFB5*	0.03	−2.42
sp|P48818	28	Very long-chain specific acyl-CoA dehydrogenase, mitochondrial	*ACADVL*	0.01	−2.42
sp|Q3T189	13	Succinate dehydrogenase iron-sulfur subunit, mitochondrial	*SDHB*	0.01	−2.42
tr|F1MMT2	62	Laminin subunit alpha 2	*LAMA2*	0.00	−2.56
sp|Q2NL34	6	Ubiquinone biosynthesis protein COQ9, mitochondrial	*COQ9*	0.04	−2.62
sp|Q95140	7	60 S acidic ribosomal protein P0	*RPLP0*	0.02	−2.94
tr|Q24JZ7	11	Succinyl-CoA:3-ketoacid-coenzyme A transferase	*OXCT1*	0.04	−3.02
tr|Q2KHU2	9	Leucine rich repeat containing 2	*LRRC2*	0.02	−3.06
tr|F1MNT4	24	Laminin subunit beta 1	*LAMB1*	0.00	−3.08
sp|P22439	11	Pyruvate dehydrogenase protein X component	*PDHX*	0.01	−3.13
sp|Q00361	5	ATP synthase subunit e, mitochondrial	*ATP5ME*	0.01	−3.40
sp|P13620	10	ATP synthase subunit d, mitochondrial	*ATP5H*	0.01	−3.73

### Protein classification by molecular function and functional enrichment analysis

Four DAPs in the R1 vs. A1 analysis mapped to biochemical pathways in Ingenuity Pathway analysis (IPA) software and these are displayed in Supplementary Fig. S1. The main biological processes affected by these DAPs included cellular development, growth and proliferation and connective tissue development. In relation to the A2 vs. A1 and the R2 vs. A2 analysis no functional enrichment was evident due to a lack of DAPs.

**Figure 2:**
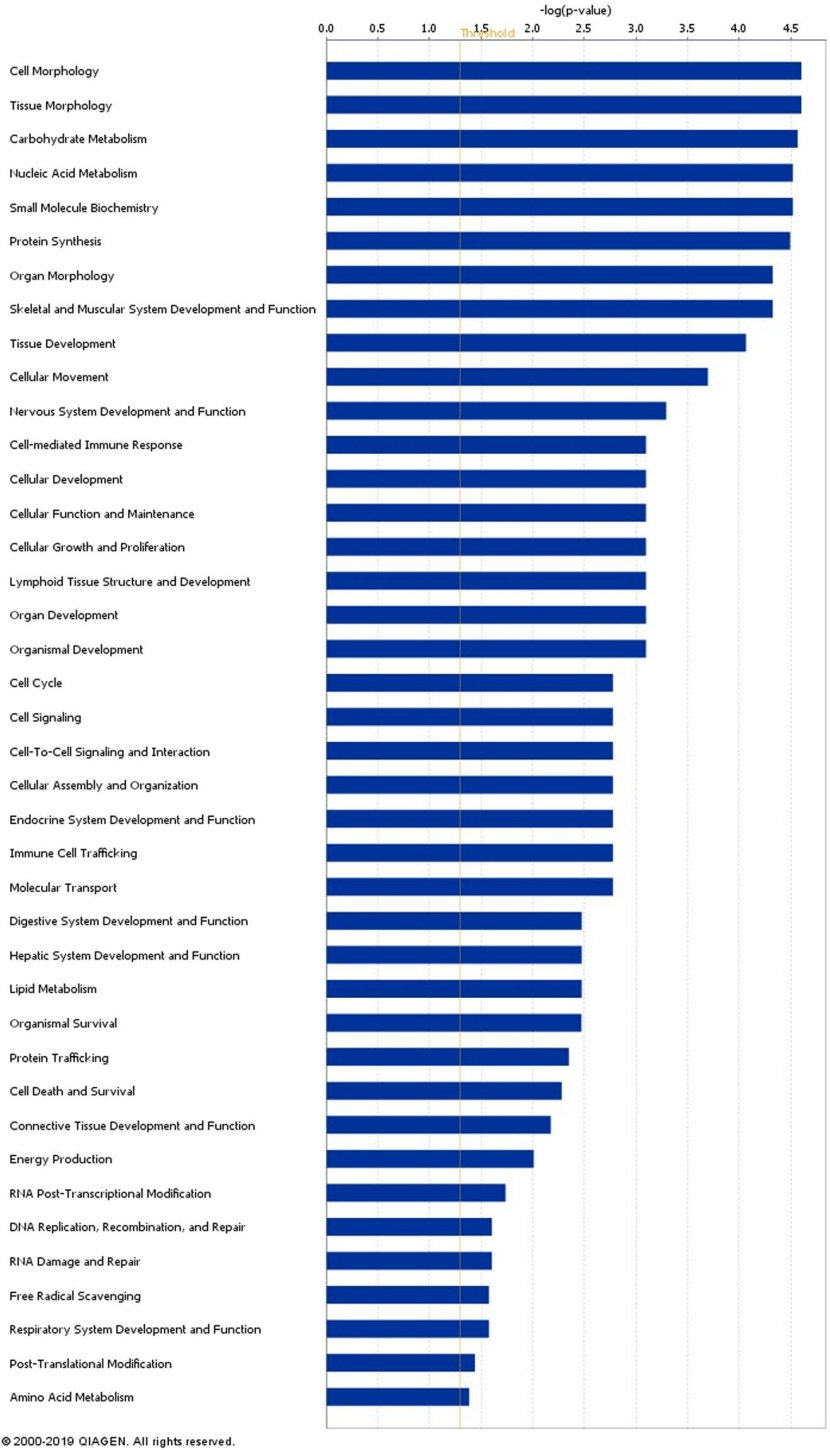
Classification of molecular and cellular functions of differentially abundant proteins in beef cattle undergoing compensatory growth compared to feed restricted cattle (R2 vs. R1). The bars indicate the likelihood [−log (P value)] that the specific function was affected by compensatory growth compared with others represented in the list of differentially abundant proteins.

In relation to the CG comparison (R2 vs. R1) 37 out of the 39 DAPs were successfully mapped to a biological or molecular pathway and/or category in the IPA database. These DAPs were analysed and assigned to their associated molecular and cellular functions (Fig. 2) within IPA. Proteins involved in cell and tissue morphology, carbohydrate metabolism and nucleic acid metabolism were differentially abundant.

**Figure 3:**
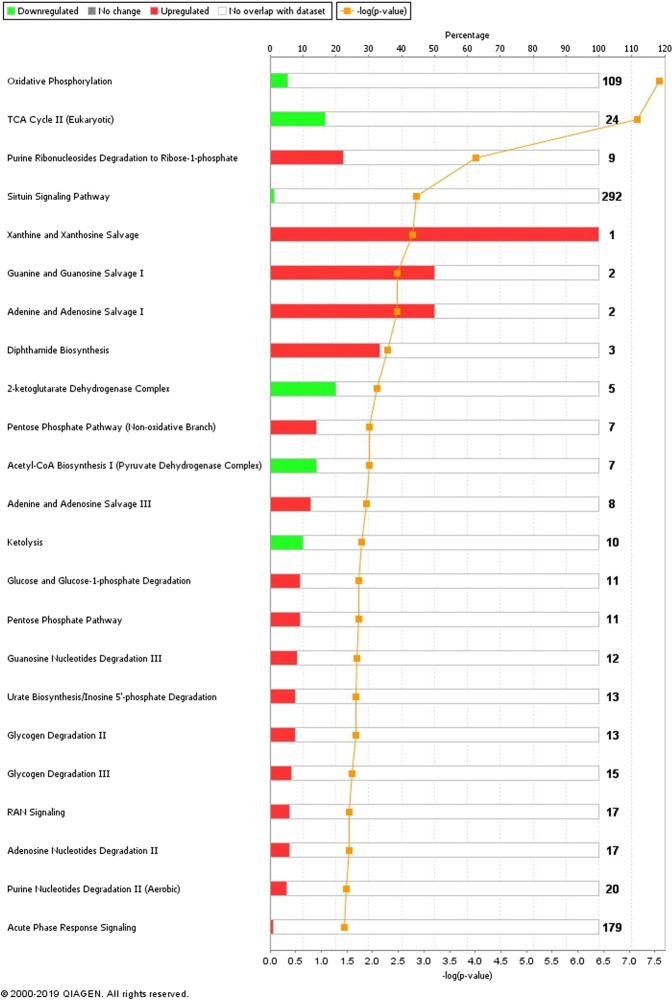
Biochemical pathways which were significantly enriched in bulls undergoing compensatory growth compared to feed restricted bulls (R2 vs. R1). Red bars represent up- regulated proteins and green bars display down-regulated proteins as an overlap of the total number of proteins which are involved in that pathway in the study dataset and the pathway as a whole. The p-value displayed is calculated by the number of proteins in the R2 vs. R1 dataset which are involved in that particular pathway divided by the total number of proteins present in that canonical pathway in the IPA knowledgebase. The yellow line [−log (p-value)] represents the significance of each pathway.

**Figure 4:**
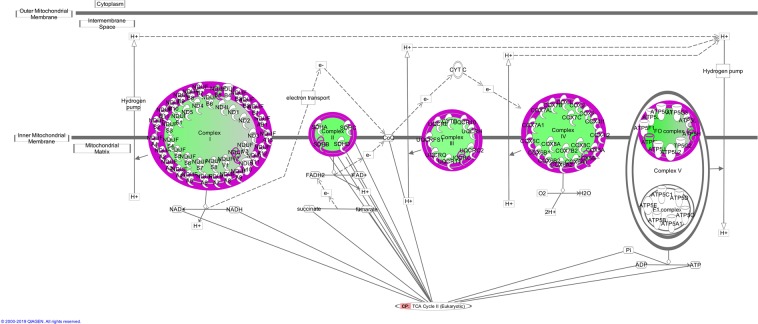
Diagram of oxidative phosphorylation pathway and the tricarboxylic acid cycle combined. These pathways were identified as two of the significant pathways by IPA. Proteins which are outlined in purple are present in our dataset of animals undergoing compensatory growth compared to feed restricted animals (R2 v R1). The inner green colour indicates these proteins are down-regulated in animals undergoing compensatory growth compared to feed restricted animals.

Figure 3 displays details of the biochemical pathways which the DAPs in the R2 versus R1 analysis are involved in. Of particular interest to the expression of CG was the downregulation of oxidative phosphorylation and TCA cycle pathways (Fig. 4). Downregulation was observed for four proteins involved in the TCA cycle (IDH3A, SDHB, DLST, ACO2) and six proteins in the oxidative phosphorylation pathway (UQCRC1, SDHB, ATP5F1A, COX5B, NDUFB5, NDUFV2) in the R2 group compared to R1.

**Figure 5:**
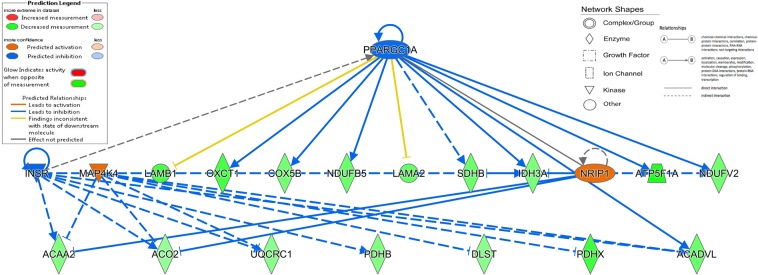
Upstream Regulator Analysis hierarchical graph which displays likely upstream regulators that are connected to differentially abundant proteins between cattle undergoing compensatory growth and cattle fed a restricted diet (R2 vs. R1) through a set of direct and indirect relationships.

Upstream analysis (Fig. 5) was also carried out in IPA and 4 biological molecules including *PPARC1A*, *MAP4K4*, *INSR* and *NIRP1*, may have affected by the abundance of proteins in our data set. Nine proteins in our data set (see Supplementary Table S1) are known to be involved in the regulation of *PPARC1A*, which was predicted to be inhibited (Z score −2.105). These 9 proteins were IDH3A, COX5B, NDUFV2, ACADVL, NDUFB5, LAMA2, OXCT1, LAMB1, and ATP5F1A. Seven of these proteins are typically up regulated by *PPARC1A* however these proteins are down-regulated in our dataset therefore *PPARC1A* is predicted to be inhibited by the direction of regulation of our identified proteins. *MAP4K4* was predicted to be activated due to differential abundance of six proteins in our dataset, namely; ACO2, DLST, UQCRC1, ACAA2, ACADVL and PDHX (see Supplementary Table S2). *INSR* is predicted to be inhibited by eight proteins identified as differentially regulated in our data set. All 8 of these proteins have a direction measurement consistent with the inhibition of *INSR* (see Supplementary Table S3). These 8 proteins are ACO2, UQCRC1, ACAA2, PDHB, IDH3A, NDUFV2, ACADVL and ATP5F1A. Four of the DAPs from our study, namely; ACO2, ACAA2, IDH3A and SDHB, are all known to be downregulated by *NRIP1*. All four of these proteins were downregulated in our analysis therefore they were predicted to be activated by *NRIP1* (see Supplementary Table S4).

### Transcriptome – proteome expression analysis

**Figure 6:**
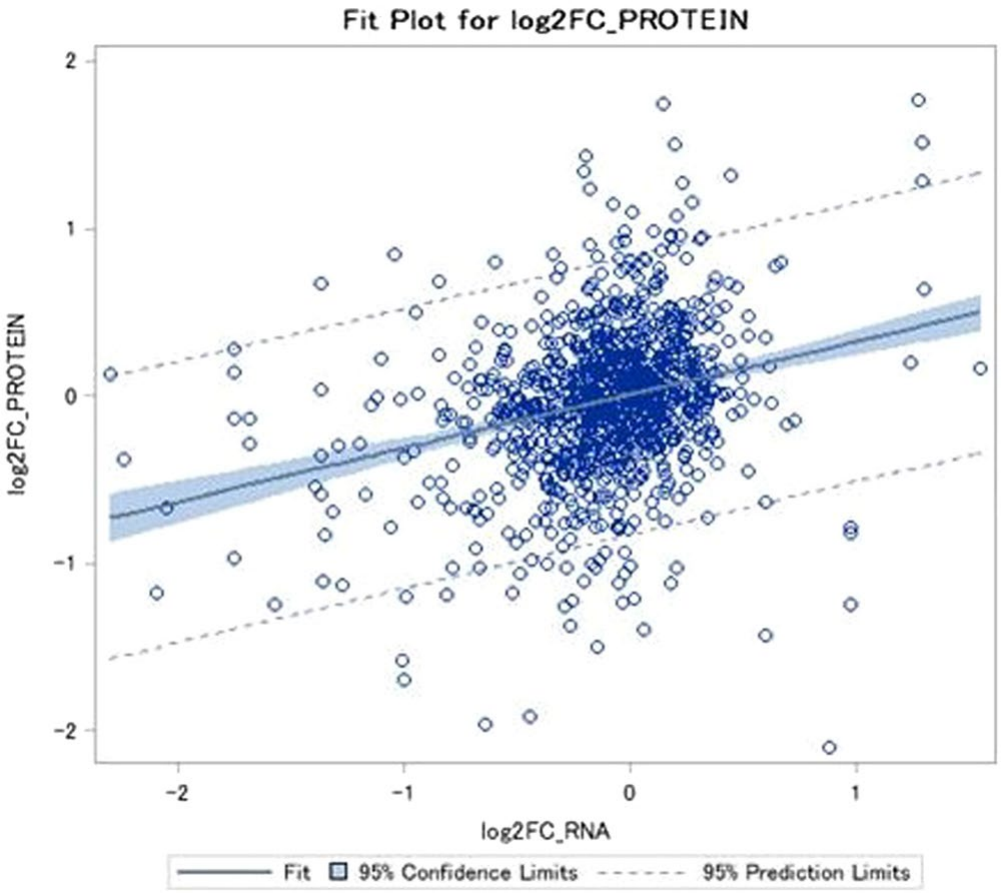
Scatterplot representing mRNA log2 fold changes versus protein log2 fold changes between Holstein Fresian bulls fed a restriction diet and Holstein Fresian bulls fed *ad libitium* for 125 days (R1 vs. A1 at the end of Period 1). n = 1104 gene-protein matches, r = 0.27, n = 1104, r^2^ = 0.075, p < 0.001. This correlation represents a general measure of how well mRNA and protein expression corresponds to each other.

Correlation analysis from transcriptome – proteome expression data at the end of Period 1 showed a low correlation coefficient between the mRNA and protein expression levels. Figure 6 presents the correlation plot (r = 0.27, n = 1104, r^2^ = 0.075, p < 0.001).

## Discussion

Compensatory growth has been incorporated into beef production systems for many years in order to reduce the lifetime feed costs of the animals produced^1,6,31^. There is clear phenotypic variation in the expression of CG; suggesting that this process is genetically controlled^32,33^. Thus there may be an opportunity to genomically select cattle with an enhanced CG potential. The objective of this study was to identify key proteins involved in the expression of CG and to determine whether the biological processes and pathways associated with CG at the mRNA previously revealed by our group^13–15^ are also relevant at the protein level. These results undoubtedly indicate a greater functional role at the phenotype level of the differentially expressed genes which have previously been identified by Keogh *et al*.^13–15^. Over the past two decades there has been major research in the area of genomics and proteomics of livestock species. Proteomic studies are an essential bridge from genome and transcriptome investigations to the production of functional proteins^34^. Mass spectral peak intensities of peptide ions have been shown to correlate well with protein abundances in complex samples^35^. As previously mentioned there were no DAP identified in the R2 vs. A2 comparison. Only one protein was found to be differentially abundant in the A2 vs. A1 analysis and 4 DAP in the R1 vs. A1 comparison. It was not surprising to observe a lack of DAP in these groups as this was consistent with results from the transcriptional gene expression studies previously conducted on these animals^13–15,17^. However although the protein and mRNA results are generally consistent with this study and the transcriptome studies of the same animals in regards to the classes of molecular and cellular functions and pathways commonly expressed between the studies during CG, the correlation coefficient between mRNA and protein abundance were not strongly correlated. This was not surprising as the Pearson correlation coefficient (r) between mRNA and protein concentrations in multi-cellular organisms have been found to be surprisingly low in the past, typically ranging from 0.09 to 0.46^22^. Therefore this discussion will focus mainly on the 39 DAPs identified in the R2 vs. R1 analysis as these are more focused on CG expression.

As already mentioned, currently there has been only one previous proteomic study examining the effect of CG on the muscle proteome^30^. This study identified a total of 26 proteins in the *longissimus thoracis* muscle. Of these 26 differentially expressed proteins; 25 were shown to have higher abundance in the continuous growth group and only one protein; Myosin-binding protein H had higher abundance in the discontinuous growth group. However although that study collected samples much later into CG compared to this study, in our own results we also observed a higher abundance of a Myosin protein; Myosin heavy chain 4 (MHY4), in the bulls undergoing CG compared to bulls fed a restricted diet (R2 vs. R1 analysis). Similarly, in the differential gene expression study of Keady (2011)^25^, on muscle tissue, *MHY4* was also found to be in higher abundance in bulls undergoing CG. Muscle samples in that study particular study were collected 4 months into re-alimentation therefore the detection of this Myosin gene could be indicative of sustained growth over a longer time period. Both of these molecules are members of the myosin family of proteins. Myosins utilise ATP to generate cellular movements and are associated with growth and increased cellular actively^36^. As well as the up-regulation of MYH4, there was also up regulation of other proteins in our DAPs related to cellular binding processes namely; TKT, SERPINI, METTL14, ANXA1, RCC1, EDF1, EEF2, SEPT2. A number of corresponding genes related to the expression of ‘binding’ proteins were also reported as differentially expressed genes (DEG) in the same cohort of cattle undergoing CG in various tissues in the studies of Keogh *et al*.^13–15^. The increase in cellular activity was also evident at the transcriptional level on day 15 of re-alimentation of CG in muscle tissue^14^. This evidence for an increase in ATP production and cellular activity coincides with the phenotypic results during this same period, whereby greater muscle growth was evident in the animals used in the current study^5^. This also coincides with greater nutrient intake. Greater cellular activity is also required to process the increased nutrient load within cells^12^. This trend of increased cellular activity has also been observed in feed efficient cattle in other studies^37,38^. Such results correspond with our own data as the feed conversion ratio (FCR) was superior in the R2 group of animals undergoing CG during Period 2 compared with *ad lib* (A1 and A2) animals over both periods. EDF1 was also identified as an up-regulated DAP in the R2 vs. R1 comparison. This protein is involved in lipid metabolism. The up-regulation of this protein concurs with phenotypic results at the end of Period 2, where R2 bulls exhibited a reduced fat covering compared to A2 bulls (fat cover scores: R2 vs. A2: 5.1 v 7.6, P < 0.05)^5^. Furthermore another protein identified in our dataset relates to cellular binding processes was also observed as differentially expressed at the transcriptome level. *SERPINH1* was also reported as a DEG in ruminal epithelial tissue^39^ (Table 3).

**Table 3:** Genes pertaining to proteins identified which were identified as commoly expressed in this study and in transcriptomic gene expression studied as differentally expressed genes during compensastory growth.

Reference	Gene name	Fold change of proteins in the current dataset (R2 vs. R1)	Fold change of genes in referenced dataset
Keogh *et al*.^14^	*RCC1*	3.37	1.28
	*PDHB*	−2.02	−1.28
	*IDH3A*	−2.14	−1.28
	*NDUFV2*	−2.35	−1.33
	*NDUFB5*	−2.42	−1.51
Keogh *et al*.^39^	*ACADVL*	−2.42	1.29
	*SERPINH1*	4.76	1.53
O Connor *et al*.^11^	*FGG*	3.29	6.13
	*PSMB7*	2.82	2.02
	*EDF1*	2.14	3.82
	*RPLP0*	−2.94	3.53
	*ACAA2*	−2.01	2.34
	*ACADVL*	−2.42	4.04

These genes may hold potential use as DNA based biomarkers for the genomic selection of compensatory growth in beef cattle.

Acetyl-CoA biosynthesis 1 (downregulated), 2-ketoglutarate dehydrogenase complex (downregulated), pentose phosphate pathway (upregulated) and ketolysis (down-regulated) were affected by CG and all form part of pathways which regulate the generation of precursors of metabolites and energy. The down regulation of these pathways during re-alimentation may have resulted in less energy production in the muscle tissue. This is a similar trend to that observed at the mRNA level with the down-regulation of genes coding for succinyl CoA synthetase which produces ATP^14^. Our results were corroborated by those reported in the study of Connor *et al*.^11^ whereby genes relating to oxidative phosphorylation were not detected or down-regulated on day 14 of re-alimentation of CG compared to the control group. Similarly in the study of Alemida *et al*.^30^ several proteins related to energy metabolism had a higher abundance in the bulls on the continuous growth path compared to the bulls on the discontinuous growth trajectory. These results together may suggest an adaptation to reduce energy production from dietary derived nutrients, leading to less energy being produced for maintenance and therefore more energy available for growth processes.

Possibly the most significant results from this study are those in relation to sirtuin signalling. The TCA cycle and the electron transport chain produce 90% cellular ATP generated in the mitochondria^40,41^. The sirtuin signalling pathway is involved in both TCA cycle and oxidative phosphorylation. Recent findings have shown that mitochondrial sirtuin signalling functions in the control of basic mitochondrial biology, including energy production, metabolism, apoptosis, and intracellular signalling^42^. Four proteins involved in the TCA cycle (IDH3A, SDHB, DLST and ACO2) and six proteins in the oxidative phosphorylation pathways (UQCRC1, SDHB, ATP5F1A, COX5B, NDUFB5, NDUFV2) were down-regulated in R2 vs. R1. These results are consistent with the transcriptional study of Keogh *et al*.^14^ in muscle tissue, which found that *IDH3A* and *NDUFB5* were both also identified as down regulated in bulls undergoing CG. Furthermore across species, the TCA cycle was also noted as inhibited in high feed efficient pigs in a proteomic study by Fu *et al*.^43^. In that study, the authors identified down-regulation of DAPs in the high feed efficient pigs (low residual feed intake) and concluded that energy metabolism in the skeletal muscle is negatively correlated with feed efficiency. These conclusions were similar to results from the work of Vincent *et al*.^44^ in pigs divergent in feed efficiency. Additionally in a study published recently by Cui *et al*.^45^ on the effects of dietary protein restriction followed by realimentation on growth performance and liver transcriptome alterations of lambs, genes involved in tyrosine and metabolism were down-regulated in the realimentation phase^45^. Overall, our study found a down-regulation in mitochondrial function in the muscle proteome of animals undergoing CG. These results have also been consistently observed at the transcriptional level during CG in beef cattle^12,14,15^. Alexandre *et al*.^46^ also reported the enrichment of oxidative-reduction in a co-expression network negativity associated with feed efficiency in liver tissue of beef cattle. Increased mitochondrial function has been traditionally associated with enhanced feed efficiency^18,47–49^. However our proteomic study and transcriptomic studies examining muscle tissue during CG^14^ indicate that this does not seem to be the case in relation to CG. As such our hypothesis for this trend during CG is that oxidative phosphorylation is down-regulated in a cell survival mechanism in order to cope with the sudden influx of extra nutrients and metabolites in the cell and increased cellular activity. This was further evident by the enrichment of three pathways involved in nucleotide biosynthesis in the R2 vs. R1 comparison. These biochemical pathways are Xanthine and Xanthosine salvage, adine and adenosine salvage 1 and guanine and guanosine salvage 1. A corresponding increase in the abundance of genes involved in growth and cellular activity during re-alimentation had been previously identified as having a contribution to the expression to CG^11,13,15^, results from our dataset show this is also the case at the protein level.

It is beneficial to identify upstream regulators such as transcriptional factors, genes or small molecules to provide biological insights in to the observed expression changes^50^. Regulators identified are predicted by IPA to be inhibited or activated based on the up or down-regulation pattern of the expressed proteins in the dataset being studied compared to what is expected from the literature and also how many known targets of each transcription regulator are present in the uploaded dataset being studied. If the observed direction of change in the proteins of our study dataset is mostly consistent with a particular activation state of the transcriptional regulator (“activated” or “inhibited”), then a prediction is made about that activation state. In the R2 vs. R1 analysis, there were four upstream regulators identified. Seven out of nine proteins in our dataset which are involved in the up regulation of *PPARC1A* had negative FCs in our study, indicating that *PPARC1A* was inhibited. The protein (PGC-1α) encoded by *PPARC1A* is a transcriptional co-activator that controls genes involved in energy metabolism and is also a major factor which regulates muscle fibre type determination^51^. The inhibition of this gene may be responsible for the down regulation of proteins in the TCA and oxidative phosphorylation pathways pertaining to metabolism. Unsurprisingly, three of the DAPs identified in the current dataset involved in the inhibition of this gene also had their corresponding genes down regulated in gene expression studies during CG in these animals. Specifically, *IDH3A*, *NDUFB5* and *NDUFV2* were identified as downregulated DEGs in muscle tissue of these animals^14^. This hypothesis may be further evidenced by the down regulation of 5 other PPAR genes in the transcriptional study on rumen tissue of the same cohort of animals^39^ and also the negative FC of 6 other PPAR genes in muscle tissue during CG^14^. *MAP4K4* was also identified as an upstream regulator by IPA. Six of our DAPs identified, namely; ACO2, DLST, UQCRC1, ACAA2, ACADVL and PDHX6 were all down regulated which is consistent with the activation of *MAP4K4*. This protein is a member of the serine/threonine protein kinase family. This family of proteins are involved in phosphorylation; the transfer of phosphates to the oxygen atom of a serine or threonine side-chain proteins^52,53^. *ACAA2* was also previously identified as a DEG in ruminal epithelial during CG in the study of Keogh *et al*.^17^. *INSR* (Insulin Receptor) was predicted to be inhibited by 8 proteins in our dataset. All 8 of these proteins had a measurement consistent with the inhibition of this upstream regulator. Typically these 8 proteins are upregulated by *INSR* however they showed a downregulated in our dataset which leads to the hypothesis that *INSR* was inhibited. During Period 2 the R2 group had an increased dry matter intake compared to their A2 counterparts^10^. This increased feed intake is associated with the an increase of glucose in the blood towards the end of Period 2 which subsequently causes the production of insulin from the pancreas^9,54^ and an increase in systemic concentrations of insulin in the blood^10^. In this study it was found that insulin receptor was inhibited suggesting a latent lower uptake of glucose into cells alternatively this may be due to insulin sensitivity sustained from the previous dietary restriction phase^55^. Furthermore two DAPs identified in our dataset as being involved in the inhibition of *INSR* were also identified as DEGs in the transcriptional gene expression studies on these animals. *IDH3A* was also identified as a DEG in the *M*. *longissums dorsi* of these animals^14^.

Compensatory growth is a multifaceted process, under the control of many biological processes. The variation in the expression of the CG trait suggests that this trait is genetically controlled. By gaining a deeper understanding of the genetic control of this economically important trait the potential exists to develop DNA based biomarkers for the genomic selection of genetically superior, energetically efficient beef cattle which hold a greater genetic predisposition to undergo CG, and thus produce beef that is more economically sustainable. Results from this study identified proteins which were differentially abundant during CG (n = 39). These proteins were involved in cellular binding processes, oxidative phosphorylation and mitochondrial function. There was also evidence for up regulation of three pathways involved in nucleotide biosynthesis. Additionally this study validates functional genes at the proteome level which may contribute to the expression of CG. A number of functional genes pertaining to proteins identified in this study may hold genetic variants with potential for use as candidates for DNA based biomarkers for genomic selection of CG. Further investigation into possible genomic variants that may be present in these genes is required.

## Materials and Methods

All procedures involving animals were approved by the University College Dublin Animal Research Ethics Committee and licensed by the Irish Department of Health and Children in accordance with the European Community Directive 86/609/EC (http://www.dohc.ie/other_health_issues/pausp).

### Animal model and tissue sampling

This study was conducted as part of a larger study designed to physiologically characterise the effect of restricted feeding and subsequent re-alimentation in Holstein Friesian (HF) bulls. The animal model utilised has previously been described in detail by Keogh *et al*.^5,10^. In brief, 40 purebred HF bulls were assigned to one of four groups: (i) restricted feed allowance for 125 days (R1); (ii) restricted feed allowance for 125 days followed by *ad lib* access to feed for a further 55 days (R2); (iii) *ad lib* access to feed for 125 days (A1); or (iv) *ad lib* access to feed for 125 days followed by a further *ad lib* feeding period of 55 days (A2). The first 125 days was denoted as Period 1 and the subsequent 55 days, Period 2. At a mean age of 485 (±14) days and weight of 369 (±31) kg all animals were individually fed (70% concentrate and 30% grass silage diet) according to their treatment group with R1 and R2 animals expected to gain 0.6 kg/day and A1 and A2 groups were expected to gain in excess of 1.5 kg/day during Period 1. All bulls were weighed regularly throughout the trial to assess their growth performance^5^. At the end of Period 1, all animals from each R1 and A1 treatment groups were slaughtered. All remaining animals (groups R2 and A2) were slaughtered at the end of Period 2. At each time-point slaughter order was randomised to account for potential confounding effects on treatment results. *M*. *longissimus dorsi* samples were collected immediately after slaughter at the end of Period 1 and on day 55 of re-alimentation in Period 2. Samples were washed in DPBS, snap frozen in liquid nitrogen and stored at −80 °C until protein extraction was performed. Tissue collection procedures are described in detail by Keogh *et al*.^14^.

### Protein extraction and quantification

From the 40 muscle tissue samples collected, 39 samples were used for this study (one sample was removed from the study due to insufficient tissue for protein extraction). These 39 samples consisted of 10 samples from each of groups ‘A1’; ‘A2’ and ‘R2’; and 9 from ‘R1’. Frozen muscle samples (100 mg) were harvested with a scalpel blade and placed in tubes containing 0.5 g of silica beads. Samples were then homogenized with a Percellys 24 homogenizer (Bertin Corp, Rockville, USA) at 2 × 20 seconds at 6500 × g with 1 ml of extraction buffer. The extraction buffer was comprised of 7 M Urea, 2 M Thiurea, 1% (w/v) DTT (Dithiothreitol), 2% (w/v) CHAPS (3-[(3-Cholamidopropyl) dimethylammonio]-1-propanesulfonate hydrate), 2% IPG buffer pH 3–10 and 1% Protease Inhibitor Mix (GE Lifesciences, Uppsala, Sweden). The samples were then transferred into a new Eppendorf tube. Samples were vortexed for 1 minute and subsequently mixed for 1 hour at room temperature using a tube rotator (VWR), following which samples were centrifuged at 10,000 × g for 10 minutes at room temperature. After centrifugation, the supernatant was transferred to a new tube and protein concentrations were subsequently determined using the 2D Quant kit (GE Lifesciences, Uppsala, Sweden) following the manufacturer’s instructions.

### Sample preparation for label-free proteomic quantification

Label-free Mass Spectrometry proteomic quantification was undertaken commercially by Functional Genomics Centre Zurich (ETH Zurich, University of Zurich, Switzerland). The isolated proteins were solubilized using High Intensity Focused Ultrasound (HIFU) for 1 minute. The ultrasonic amplitude for HIFU was set to 65%. The solubilised proteins were then diluted with water in a 1:10 dilution following which the protein concentrations were calculated using the Qubit® Protein Assay Kit (Life Technologies, Zurich, Switzerland). Samples were then prepared using a commercial iSt Kit (PreOmics, Germany). A revised version of the standard protocol was used. In brief, 100 µg of protein was solubilized in the ‘Lyse’ buffer. This solution was then boiled at 95 °C for 10 minutes, after which it was subsequently processed with HIFU for 30 seconds. The ultrasonic amplitude for HIFU was set to 85%. After processing with HIFU the resulting solution was then transferred to the cartridge. The proteins were then digested by adding 50 µl of the ‘Digest’ solution and the samples were then incubated with the ‘digest’ solution at 37 °C for 60 minutes following which 100 µl of ‘Stop’ solution was added to stop the digestion process. The peptides in the cartridge were retained by the iST-filter and the solutions were removed by centrifuging at 3800 x g. Lastly, the resulting peptides were washed, eluted, dried and re-solubilised in 20 µl of buffer containing 3% acetonitrile and 0.1% formic acid for subsequent Liquid Chromotrophy (LC) -Mass Spectrometry (MS) analysis.

### Liquid chromatography - mass spectrometry analysis

All MS analyses were performed in a random order on a Q Exactive HF-X mass spectrometer (Thermo Scientific) equipped with a Digital PicoView source (New Objective, USA) and attached to an M-Class Ultra Performance Liquid Chromatographer (Waters, Milford, MA, USA). The composition of the solvent at both channels (A and B) was made up of 0.1% formic acid for channel A and 0.1% formic acid, 99.9% acetonitrile for channel B. 2 μL of peptides from each sample were loaded on a commercial MZ Symmetry C18 Trap Column (100Å, 5 µm, 180 µm × 20 mm, Waters Corporation, MA, USA) and then loaded onto a nanoEase MZ C18 HSS T3 Column (100Å, 1.8 µm, 75 µm × 250 mm, Waters). Peptides were eluted by a gradient from 8 to 27% at a flow rate of 300 nL/min in 82 min; 35% in 5 min and 80% in 1 min. The samples were acquired in a randomized order. Data-dependent mode was selected when operating the mass spectrometer. This gave a full-scan of MS spectra (350−1’400 m/z) at a resolution of 120,000 at 200 m/z after accumulation to a target value of 3,000,000. This was followed up by fragmentation on the twenty most intense signals per cycle. Using normalized collision energy of 28 and a maximum injection time of 22 ms., higher-energy collision dissociation spectra were acquired at a resolution of 15,000. The automatic gain control was set to 100,000 ions. Charge state screening was set to singular and unassigned and any charge greater than seven were rejected. When selected for MS/MS, only precursors with intensity above 110,000 were selected. Any precursor masses which were previously selected for MS/MS measurement were excluded from further selection for 30 seconds. The exclusion window was set at 10 ppm. The samples were acquired using internal lock mass calibration on m/z 371.1012 and 445.1200.

### Proteomic data analysis

The raw data from MS was subsequently processed in MaxQuant (version 1.6.2.3). Protein identification was then carried out using the integrated Andromeda search engine^56^. Resulting spectra were scanned against a Uniprot *Bos Taurus* reference proteome, (taxonomy 9913, canonical version from 17-08-2017) concatenated to its reversed decoyed FASTA database and common protein contaminants. Methionine oxidation and N-terminal protein acetylation were both set as variable and Carbamidomethylation of cysteine was set as a fixed modification. A minimal peptide length of 7 amino acids and a maximum of two missed-cleavages were allowed with enzyme specificity set to trypsin/P. The MaxQuant Orbitrap default search settings were used. For peptides the maximum false discovery rate (FDR) was set to 0.01 and 0.05 for proteins. A two minute window for match between runs was set when label free quantification was enabled. Each file was kept separate in the MaxQuant experimental design template, to obtain individual quantitative values.

For protein quantification, four experimental comparisons were used: i) R1 (Restricted feeding for 125 days) vs. A1 (*ad lib* feeding for 125 days); ii) R2 (Restricted feeding for 125 days and *ad lib* feeding for 55 days); vs. A2 (*ad lib* feeding for 125 and 55 days); iii) A2 vs. A1; iv) R2 vs. R1. The two comparisons containing the ‘R2’ treatment group, namely; R2 vs. A2 and R2 vs. R1 are the most focal for this study as the R2 treatment group underwent CG. The R1 vs. A1 comparison was included to examine any proteome changes due to diet variation in Period 1 and also to correlate protein and available mRNA abundance data from the same animals at the end of Period 1. The A2 vs. A1 analysis examined any potential time effect in *ad lib* fed animals across the two periods. For each comparison, proteins were considered differentially abundant if a minimum of 2 peptides per protein were quantified, the Bonferroni p-value was < 0.05 and a FC > 1.5 or < −1.5 was apparent between the comparisons. Protein FCs were computed based on intensity values following label-free quantification. A positive fold change indicates that the protein in the first group is upregulated compared to the second group. A negative fold change indicates that the protein in the first group is downregulated compared to the second group.

Using the R package SRMService, a set of functions was implemented to normalize the data with a modified robust z-score transformation and to compute p-values using parametric t-test with pooled variance and also to filter for proteins with 2 or more peptides allowing for a maximum of 5 missing values. If all measurements of a protein were missing in one of the conditions, a pseudo FC was calculated replacing the missing group average by the mean of 10% smallest protein intensities in that condition.

The mass spectrometry proteomics data were handled using the local laboratory information management system^57^. All relevant data has been deposited to the ProteomeXchange Consortium via the PRIDE (http://www.ebi.ac.uk/pride) partner repository^58^ with the data set identifier PXD014374.

### Protein characterisation and functional enrichment analysis

Functional enrichment analysis was performed using Ingenuity pathway analysis (IPA) (Ingenuity Systems, Redwood City, CA; http://www.ingenuity.com), a web-based software application which allows analysis of over-represented biological mechanisms, biochemical pathways and upstream regulators in genes of interest^50^. Inputs used in the Pathway Analysis software included Ensembl gene identifiers, FCs and P values for DAPs.

### Transcriptome-proteome expression correlation analysis

Protein abundance data from the current study and mRNA expression data previously published by Keogh *et al*.^14^ from the same animal model were used to determine the relationship between expressed proteins and expressed transcripts at the end of Period 1. Detected genes (n = 18488) from the RNA seq data set of Keogh *et al*.^14^ at the end of Period 1 (RES v ADLIB)^14^ were matched by Ensembl gene IDs against detected proteins (n = 1142) for the same cohort of animals from the R1 vs. A1 proteomic study, resulting in a total of 1104 gene-protein matches.

Pearson correlation coefficients were calculated between gene-protein matches using Statistical Analysis Software (SAS Inst. Inc., Cary, NC). Coefficients were correlated by the log2 FC values for mRNA and protein expression at the end of Period 1. Transcriptome-proteome expression correlation analysis was only carried out for the R1 vs. A1 comparison at the end of Period 1 as this was the only time point where both proteomics and transcriptome sequencing was undertaken simultaneously.

## Supplementary information

Supplementary material.

## Data Availability

The datasets analysed in the current study are available on the ProteomeXchange Consortium via the PRIDE (http://www.ebi.ac.uk/pride) partner repository with the data set identifier PXD014374.
