# Supplementary material for: Label-free quantitative proteomic analysis of M. longissimus dorsi from cattle during dietary restriction and subsequent compensatory growth
Source: Sci Rep. 2020 Feb 13;10:2613. doi: 10.1038/s41598-020-59412-6 (PMC7018817; doi:10.1038/s41598-020-59412-6)
Supplement: Supplementary file 1 — Supplementary material. [file 41598_2020_59412_MOESM1_ESM.docx]

# Supplementary material

**Label-free quantitative proteomic analysis of *M. longissimus dorsi* from cattle during dietary restriction and subsequent compensatory growth**

Authors: Yvonne Mullins^1,2^, Kate Keogh^1^ , David A. Kenny^1^ , Alan Kelly^2^, Padraig O’ Boyle³ and Sinéad M. Waters^1*^

^1^Animal and Bioscience Research Department, Animal and Grassland Research and Innovation Centre, Teagasc, Grange, Dunsany, Co. Meath. Ireland.

^2^School of Agriculture and Food Science, University College Dublin, Belfield, Dublin 4, Ireland.

³Animal and Bioscience Research Department, Animal and Grassland Research and Innovation Centre, Teagasc, Athenry, Co.Galway, Ireland.

*corresponding author: Sinéad M. Waters [Sinead.waters@teagasc.ie](mailto:Sinead.waters@teagasc.ie)


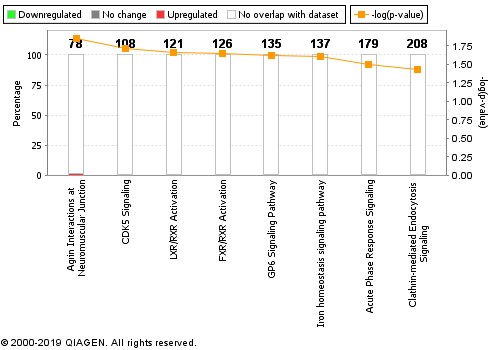


Supplementary Fig. S1: Biochemical pathways which were significantly enriched in the restricted animals in Period 1 compared to *ad libitum* fed bulls in Period 1 (R1 v A1). Red bars represent up- regulated proteins and green bars display down-regulated proteins as a percentage of the overall proteins in that pathway. The p-value displayed is calculated by the number of proteins in the R1 v A1 dataset which are involved in that particular pathway divided by the total number of proteins present in that canonical pathway in IPA. The yellow line [-log (p-value)] represents the significance of each pathway.

Supplementary Table S1: Upstream regulation of *PPARGC1A* as identified by IPA. Nine differentally abundant proteins identified in cattle undergoing compensatory growth compared to cattle fed a restricted diet for 125 days are known to be involved in the regulation of *PPARC1A*. *PPARC1A* is was predicted to be inhibited (Z score -2.105). Seven of these proteins are typically up regulated by *PPARC1A* however these proteins are down-regulated in our dataset therefore PPARC1A is predicted to be inhibited by the direction of regulation of our identified proteins.

| Ensembl Gene ID | Proteins in dataset | Prediction (based on measurement direction) | Fold change | Positive fold change would result in |
| --- | --- | --- | --- | --- |
| ENSBTAG00000006227 | IDH3A | Inhibited | -2.14 | Upregulation |
| ENSBTAG00000018542 | COX5B | Inhibited | -2.27 | Upregulation |
| ENSBTAG00000004871 | NDUFV2 | Inhibited | -2.35 | Upregulation |
| ENSBTAG00000003072 | ACADVL | Inhibited | -2.42 | Upregulation |
| ENSBTAG00000002463 | NDUFB5 | Inhibited | -2.42 | Upregulation |
| ENSBTAG00000010229 | LAMA2 | Affected | -2.56 | Regulation |
| ENSBTAG00000033186 | OXCT1 | Inhibited | -3.02 | Upregulation |
| ENSBTAG00000011412 | LAMB1 | Activated | -3.08 | Upregulation |
| ENSBTAG00000002507 | ATP5F1A | Inhibited | -3.73 | Upregulation |

Supplementary Table S2: Upstream regulation of *MAP4K4* as identified by IPA. Six differentally abundant proteins identified in cattle undergoing compensatory growth compared to cattle fed a restricted diet for 125 days are known to be involved in the regulation of *MAP4K4. MAP4K4* was predicted to be activated due to differential abundance of six proteins in our dataset these proteins are down-regulated in our dataset therefore *MAP4K4* is predicted to be activated by the direction of regulation of our identified proteins.

| Ensembl Gene ID | Proteins in dataset | Prediction (based on measurement direction) | Fold change | Positive fold change would result in |
| --- | --- | --- | --- | --- |
| ENSBTAG00000006429 | ACO2 | Activated | -1.73 | Downregulation |
| ENSBTAG00000006463 | DLST | Activated | -1.90 | Downregulation |
| ENSBTAG00000019096 | UQCRC1 | Activated | -1.93 | Downregulation |
| ENSBTAG00000002863 | ACAA2 | Activated | -2.01 | Downregulation |
| ENSBTAG00000003072 | ACADVL | Activated | -2.42 | Downregulation |
| ENSBTAG00000018261 | PDHX | Activated | -3.13 | Downregulation |

Supplementary Table S3: INSR Upstream regulation of as *INSR* identified by IPA. Eight differentally abundant proteins identified in cattle undergoing compensatory growth compared to cattle fed a restricted diet for 125 days are known to be involved in the regulation of *INSR. INSR* is was predicted to be inhibited (Z score -2.8). Eight of these proteins have a directional measurement consistant with the inhibition of *INSR*. These 8 proteins are all known to be upregulated by *INSR* and are downregulated in out datasettherefore *INSR* is predicted to be inhibited.

| Ensembl Gene ID | Proteins in dataset | Prediction (based on measurement direction) | Fold change | Positive fold change would result in |
| --- | --- | --- | --- | --- |
| ENSBTAG00000006429 | ACO2 | Inhibited | -1.73 | Upregulation |
| ENSBTAG00000019096 | UQCRC1 | Inhibited | -1.93 | Upregulation |
| ENSBTAG00000002863 | ACAA2 | Inhibited | -2.01 | Upregulation |
| ENSBTAG00000021724 | PDHB | Inhibited | -2.02 | Upregulation |
| ENSBTAG00000006227 | IDH3A | Inhibited | -2.14 | Upregulation |
| ENSBTAG00000004871 | NDUFV2 | Inhibited | -2.35 | Upregulation |
| ENSBTAG00000003072 | ACADVL | Inhibited | -2.42 | Upregulation |
| ENSBTAG00000002507 | ATP5F1A | Inhibited | -3.73 | Upregulation |

Supplementary Table S4: : Upstream regulation of as *NRIP1* identified by IPA. Four differentally abundant proteins identified in cattle undergoing compensatory growth compared to cattle fed a restricted diet for 125 days are known to be involved in the regulation of *NRIP1. NRIP1* is was predicted to be activated. All four of these proteins have a directional measurement consistant with the activation of *INSR*. These 8 proteins are all known to be downregulated by *INSR* and are downregulated in out datasettherefore *INSR* is predicted to be activated.

| Ensembl Gene ID | Proteins in dataset | Prediction (based on measurement direction) | Fold change | Positive fold change would result in |
| --- | --- | --- | --- | --- |
| ENSBTAG00000006429 | ACO2 | Activated | -1.73 | Downregulates |
| ENSBTAG00000002863 | ACAA2 | Activated | -2.01 | Downregulates |
| ENSBTAG00000006227 | IDH3A | Activated | -2.14 | Downregulates |
| ENSBTAG00000008314 | SDHB | Activated | -2.42 | Downregulates |
